# Evaluation of Load-To-Strength Ratios in Metastatic Vertebrae and Comparison With Age- and Sex-Matched Healthy Individuals

**DOI:** 10.3389/fbioe.2022.866970

**Published:** 2022-08-05

**Authors:** Dennis E. Anderson, Michael W. Groff, Thomas F. Flood, Brett T. Allaire, Roger B. Davis, Marc A. Stadelmann, Philippe K. Zysset, Ron N. Alkalay

**Affiliations:** ^1^ Department of Orthopedic Surgery, Center for Advanced Orthopedic Studies, Beth Israel Deaconess Medical Center and Harvard Medical School, Boston, MA, United States; ^2^ Department of Neurosurgery, Brigham and Women’s Hospital, Boston, MA, United States; ^3^ Department of Radiology, Brigham and Women’s Hospital, Boston, MA, United States; ^4^ Department of Medicine, Beth Israel Deaconess Medical Center and Harvard Medical School, Boston, MA, United States; ^5^ ARTORG Center for Biomedical Engineering Research, University of Bern, Bern, Switzerland

**Keywords:** spine, metastatic disease, musculoskeletal models, vertebral strength, load-to-strength ratio

## Abstract

Vertebrae containing osteolytic and osteosclerotic bone metastases undergo pathologic vertebral fracture (PVF) when the lesioned vertebrae fail to carry daily loads. We hypothesize that task-specific spinal loading patterns amplify the risk of PVF, with a higher degree of risk in osteolytic than in osteosclerotic vertebrae. To test this hypothesis, we obtained clinical CT images of 11 cadaveric spines with bone metastases, estimated the individual vertebral strength from the CT data, and created spine-specific musculoskeletal models from the CT data. We established a musculoskeletal model for each spine to compute vertebral loading for natural standing, natural standing + weights, forward flexion + weights, and lateral bending + weights and derived the individual vertebral load-to-strength ratio (LSR). For each activity, we compared the metastatic spines’ predicted LSRs with the normative LSRs generated from a population-based sample of 250 men and women of comparable ages. Bone metastases classification significantly affected the CT-estimated vertebral strength (Kruskal–Wallis, *p* < 0.0001). Post-test analysis showed that the estimated vertebral strength of osteosclerotic and mixed metastases vertebrae was significantly higher than that of osteolytic vertebrae (*p* = 0.0016 and *p* = 0.0003) or vertebrae without radiographic evidence of bone metastasis (*p* = 0.0010 and *p* = 0.0003). Compared with the median (50%) LSRs of the normative dataset, osteolytic vertebrae had higher median (50%) LSRs under natural standing (*p* = 0.0375), natural standing + weights (*p* = 0.0118), and lateral bending + weights (*p* = 0.0111). Surprisingly, vertebrae showing minimal radiographic evidence of bone metastasis presented significantly higher median (50%) LSRs under natural standing (*p* < 0.0001) and lateral bending + weights (*p* = 0.0009) than the normative dataset. Osteosclerotic vertebrae had lower median (50%) LSRs under natural standing (*p* < 0.0001), natural standing + weights (*p* = 0.0005), forward flexion + weights (*p* < 0.0001), and lateral bending + weights (*p* = 0.0002), a trend shared by vertebrae with mixed lesions. This study is the first to apply musculoskeletal modeling to estimate individual vertebral loading in pathologic spines and highlights the role of task-specific loading in augmenting PVF risk associated with specific bone metastatic types. Our finding of high LSRs in vertebrae without radiologically observed bone metastasis highlights that patients with metastatic spine disease could be at an increased risk of vertebral fractures even at levels where lesions have not been identified radiologically.

## 1 Introduction

Vertebral bone metastases are highly prevalent in cancer patients (Siegel et al., 2012), affecting 30%–50% of patients at advanced cancer stages (Sutcliffe et al., 2013). Pathologic vertebral fractures (PVFs), afflicting 15–20% of patients with spinal bone metastases (Prasad and Schiff, 2005), occur when the disease burden diminishes the strength and anatomical integrity of the affected vertebra such that it is unable to withstand daily loads. Although systemic therapy and local radiation may halt tumor progression, these treatment methods do not restore vertebral strength (Maranzano and Latini, 1995; Hartsell et al., 2005; Eleraky et al., 2010). Up to 50% of patients with PVF experience neurological deficits (Walls et al., 1995), shorter patient survival (Oefelein et al., 2002; Saad et al., 2007; Oster et al., 2013), and a lower 3-year life expectancy (Oefelein et al., 2002; Pond et al., 2014). Hence, the risk of PVF is a critical determinant in managing cancer patients with metastatic spine disease (Siegel et al., 2012; Yao et al., 2017). The spinal instability neoplastic score (SINS) (Fourney et al., 2011), which categorizes the degree of spinal instability, is widely used to predict the need for surgical stabilization in this patient population. However, the prognostic utility of SINS for predicting PVF (Cunha et al., 2012; Thibault et al., 2014) has generated conflicting reports. Sahgal et al. (2013) reported that baseline PVF, lytic tumor, and misalignment were predictive of PVF risk among the six SINS criteria. However, the SINS overall score was not predictive of PVF risk, which was also reported by Germano et al. (2016). A recent meta-analysis (Kim et al., 2021) suggested that higher SINS scores moderately predict PVF in patients after radiotherapy with a pooled sensitivity of 0.790 (95% CI 0.723–0.843) and a pooled specificity of 0.546 (0.462–0.62). Given the significant challenges of quantifying the risk of PVF clinically (Fourney et al., 2011; Boehling et al., 2012), better fracture risk prediction represents an important and unmet medical need (Galasko, 1986; Aebi, 2003; Bartanusz and Porchet, 2003; Groot et al., 2006).

Metastases permeation of the vertebral bone disrupts cellular bone homeostasis (Wu et al., 2018), resulting in remarkable changes to the bone microarchitecture (Tamada et al., 2005; Nazarian et al., 2008). Radiologically, these changes appear as osteosclerotic (bone-forming), osteolytic (bone-destroying) (Sutcliffe et al., 2013), or a combination of both, termed mixed lesions. From a mechanical perspective, PVF is caused when the metastatic lesion has degraded the vertebral strength such that it can no longer sustain loads of daily living. The mechanical strength of the vertebral bone is strongly related to the bone’s intrinsic material properties (bone matrix and mineral composition), its apparent density (the mass of bone present in the volume of interest) (McBroom et al., 1985; Mosekilde and Danielsen, 1987; Tabensky et al., 1996), and its architecture (the geometric distribution of the mass) (Kleerekoper et al., 1985; Keaveny and Hayes, 1993). In vertebrae with osteolytic bone lesions, lower vertebral bone fraction due to rarefication of bone microarchitecture and creation of lytic foci (Hojjat et al., 2011; Hojjat et al., 2012; Burke et al., 2017) and apparent loss of bone mineral content (Burke et al., 2017; Stadelmann et al., 2020) initiate the degradation of vertebral structural integrity (Hojjat et al., 2012), resulting in lower vertebral strength (Whealan et al., 2000; Alkalay, 2015; Stadelmann et al., 2020) and stiffness (Stadelmann et al., 2020). By contrast, in vertebrae containing osteosclerotic and mixed bone lesions, higher apparent bone mineral content, higher bone tissue fraction, and changes in bone trabecular bone microarchitecture (Tamada et al., 2005; Nazarian et al., 2008; Stadelmann et al., 2020) were associated with higher vertebral strength (Stadelmann et al., 2020). Vertebral loading varies greatly according to the individual’s body weight (Ghezelbash et al., 2016), spine curvature (Bruno et al., 2012; Bruno et al., 2017a; Bruno et al., 2017b), and vertebral level (Bruno et al., 2017a). Thus, it is crucial to consider vertebral strength and the loading encountered by the affected vertebra to develop a coherent assessment of fracture risk applicable across various patients and vertebral levels.

The trunk and abdominal muscles produce the majority of loading borne by the vertebral levels (Arjmand and Shirazi-Adl, 2006); the result of the need to balance the applied external loads generates the motion required by the specific task (Martuscello et al., 2013) and provides mechanical stability to maintain spinal posture (Cholewicki et al., 1997; McGill et al., 2003). However, current radiographic- (Taneichi et al., 1997) or classification (Fourney et al., 2011)-based clinical prognostic protocols used to predict fracture risk do not include direct trunk muscle measures nor consider the loads sustained. Due to the difficulty of performing spinal loading measurements *in vivo*, *in silico* biomechanical models have been created to predict spinal loading throughout the thoracolumbar spine (Han et al., 2012; Bruno et al., 2017a; Bruno et al., 2017b). A recent study by Mokhtarzadeh et al. (2021) employing a biomechanical modeling approach identified several activities that commonly cause large loads in the spine, highlighting that these activities create differing loads in different spinal regions. As metastases and PVF may occur throughout the spine, a better understanding of the pattern of vertebral loading at each specific level for common daily tasks is useful in algorithms for predicting fracture risk.

While it is well understood that PVF is a common clinical occurrence in patients with metastatic spinal disease (Kanis et al., 1999; Oefelein et al., 2001; Jensen et al., 2011), the role of spinal loading in PVF remains unstudied. Older adults with prevalent osteoporotic vertebral fractures (OVFs) have higher ratios of spinal loading to vertebral strength, or load-to-strength ratios (LSRs), compared with older adults without fractures (Duan et al., 2006; Melton et al., 2007; Melton et al., 2010; Anderson et al., 2014). Similarly, higher LSRs predict incident OVF (Wang et al., 2012; Kopperdahl et al., 2014). We have previously reported that holding a weight in flexed and upright postures produces high LSRs in the thoracolumbar region of the spine (Bruno et al., 2017a; Mokhtarzadeh et al., 2021), where OVF occurs most frequently, suggesting that the pattern of spinal loading is an important consideration in OVF (Wang et al., 2012; Kopperdahl et al., 2014). As a metastatic lesion is specific to a vertebral level and can degrade bone integrity far more rapidly than osteoporosis, PVF risk prediction requires a level- and activity-specific approach to evaluate spinal loading. Thus, the overall goal of this study is to provide the first examination of spinal loading versus vertebral strength for vertebrae with spinal metastases. Specifically, we aim to 1) validate the use of CT-based estimates of vertebral strength for vertebrae with spinal metastases, 2) evaluate LSRs for key activities, 3) examine how LSRs for vertebrae from spines with metastatic disease compared to norms in a healthy population, and 4) examine whether LSRs for metastatic vertebrae from spines with the disease vary with metastatic bone classifications, including osteolytic, osteosclerotic, and mixed lesions, and no lesions observed.

## 2 Methods

### 2.1 Specimens

**FIGURE 1:**
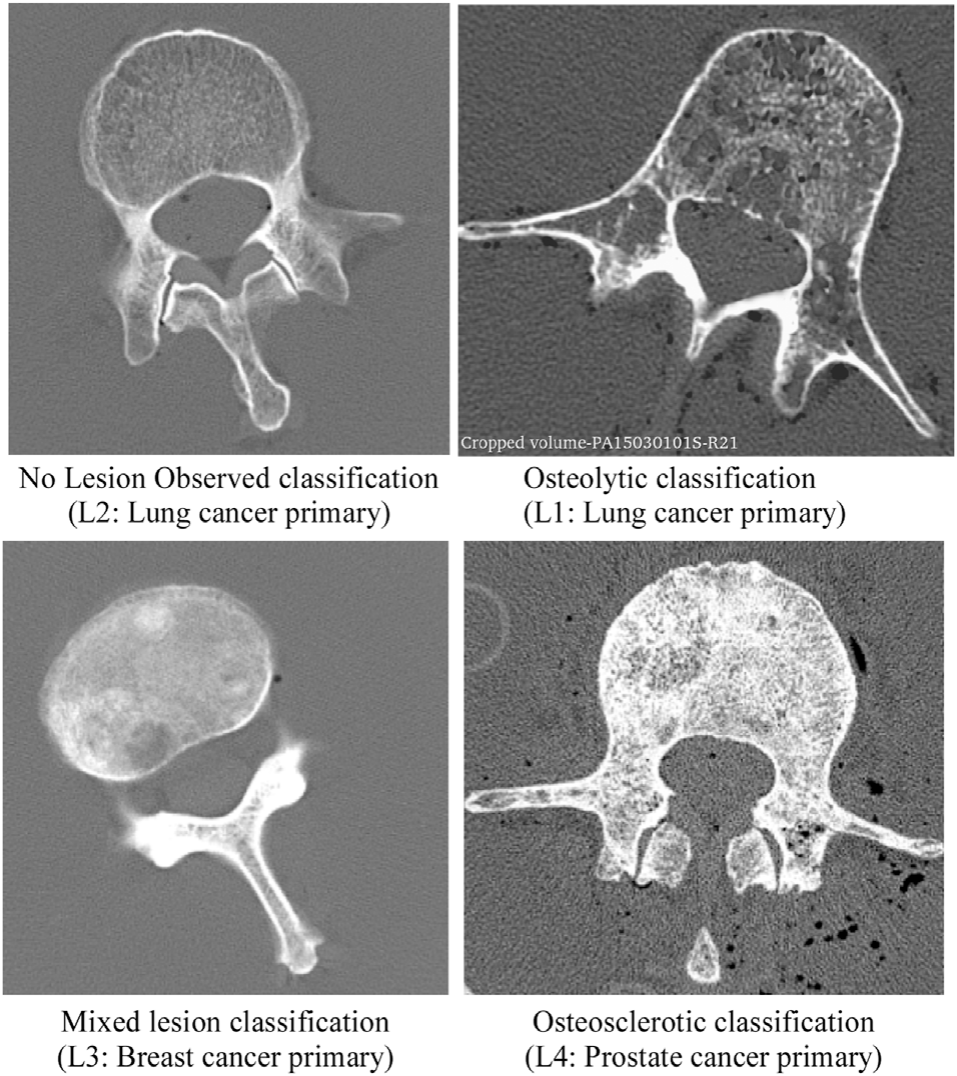
CT images presenting the radiographic appearance of vertebrae classified in this study as no lesion observed (NOL), osteolytic, mixed lesion, and osteosclerotic. The osteolytic vertebra exhibits several osteolytic foci and rarefication of bone trabecula within the vertebra. Note the destruction of bone architecture at the pedicle and transverse processes. The vertebra with osteosclerotic metastases shows unorganized bone trabeculae remodeling with high osteoid material deposition within the axial cross-section. In the vertebra with mixed metastases, several regions of sclerotic bone and large lytic foci are observed within the vertebral cross-section.

As part of a previous study by Stadelmann et al. (2020), we obtained 11 thoracolumbar cadaver spines (3 females, 8 males, age 49–71 years, mean age 54 years; Anatomy Gifts Registry, Hanover, MD) from individuals with a history of breast, lung, prostate, kidney, or esophageal cancer. Each intact spine underwent CT imaging (Aquilon 64, Toshiba Medical, United States) in a supine configuration using a custom-made imaging chamber filled with saline and a standard spine protocol (125 kV, matrix: 512 × 512, slice thickness: 0.5 mm, field of view: 16 cm), yielding an image voxel of (0.31 × 0.31 × 0.5) mm. Based on a radiological review by a neurosurgeon and neuroradiologist, each vertebral level within the spine CT dataset was classified by agreement for the presence of metastatic lesions, yielding a classification of osteolytic, osteosclerotic, mixed, or no lesion observed (NOL). Axial CT images corresponding to each bone lesion classification are illustrated in Figure 1. Demographic characteristics of metastatic spine donors are detailed in Table 1.

**TABLE 1:** Demographic characteristics of metastatic spine donors included in the study.

Spine #	Primary cancer	Age (yrs)	Race	Sex (M/F)	Height (cm)	Body mass (kg)
1	Breast	59	C	F	170.2	79.4
2	Breast	60	C	F	165.1	60.0
3	Breast	60	C	F	162.6	40.1
4	Esophageal	52	C	M	170.2	127.0
5	Kidney	71	C	M	170.2	54.4
6	Kidney	56	C	M	180.3	79.4
7	Lung	49	B	M	177.8	68.0
8	Lung	60	B	M	182.8	81.6
9	Lung	53	C	M	175.3	85.7
10	Prostate	55	C	M	182.9	68.0
11	Prostate	71	C	M	188.0	68.0

C: caucasian, B: African American. F: female, M: male.

### 2.2 Mechanical Testing

#### 2.2.1 Specimen Preparation

The measurement of 45 thoracic and lumbar levels, selected based on their lesion presentation, was detailed previously (Stadelmann et al., 2020). For each spine, the identified vertebral levels were cleaned of all musculature and ligament tissues, and the level was separated by sectioning through the disc and spinal ligaments. Per our laboratory protocol (Dall’Ara et al., 2010), a diamond blade saw (Exakt 300, EXAKT Technologies, Germany) was used to section the posterior elements proximal to the vertebral body along the vertebral coronal plane. An alignment jig was used to section both endplates under constant water irrigation, resulting in a planoparallel vertebral body specimen.

#### 2.2.2 Testing Protocol

The prepared segment was secured to a custom compression test device (Stadelmann et al., 2020) with a servo-hydraulic system (858 Mini Bionix II, MTS, Eden Prairie, United States) used to apply a monotonically increasing compressive displacement at a rate of 5 mm/min until vertebral failure was registered. Vertebral strength was defined as 1) the maximum compressive force recorded on the displacement-force curve or 2) the maximum safe force (15 kN) for the MTS built-in load cell (model: 662.20D-04).

### 2.3 Estimation of Vertebral Strength

#### 2.3.1 Finite Element prediction

In brief, each vertebral segment was scanned at an isotropic voxel size of 24.5 µm in a µCT scanner (µCT 100, Scanco Medical, Switzerland, tube voltage: 70 kV, tube current: 200 μA, integration time: 500 ms) (Stadelmann et al., 2020). A Gaussian filter (sigma: 0.8, support: 1) was applied to reduce the noise (Chevalier et al., 2009). Negative Hounsfield unit (HU) values caused by air bubbles within the trabecular space were clipped to avoid negative bone mineral density (BMD) values, and the images were down-sampled to approximately 1-mm isotropic voxel size, mimicking clinical CT image resolution. The voxels were converted into eight-noded hexahedral finite elements. The elements were assigned a local bone volume fraction (BV/TV), computed by dividing the element BMD with the mean tissue BMD of the full volume calculated in the high-resolution images (684 mgHA/cm^3^). Each element was then assigned fixed transverse isotropy with the main direction along the craniocaudal axis of the vertebra (fabric eigenvalues: 1.249, 0.894, 0.894) (Chevalier et al., 2009).

An anisotropic, time-dependent, homogenized, BV/TV-based constitutive law including linear elasticity, yielding, and plasticity with the viscous accumulation of damage and irreversible strains was applied (Pahr et al., 2014). All material constants were set to the values defined in a previous study (Pahr et al., 2014). We replicated the experimental test boundary conditions by a) fully encastering the caudal surface nodes of the vertebral segment and b) allowing the vertebral cranial surface nodes to rotate around a virtual ball joint, simulating the mechanism by which the force was applied to the vertebra. The loading conditions were prescribed as uniform axial displacement at a rate of 5 mm/min applied at the virtual ball joint. The resulting models were solved using six 3.2-GHz cores yielding about 30 min per case. The outcome of each simulation was a predicted value of vertebral strength (Fmax hFE).

#### 2.3.2 CT-Based prediction

A semi-automated image analysis program (SpineAnalyzer, Optasia Medical, Cheadle, United Kingdom) was employed to identify the mid-height of the vertebrae. The axial CT image corresponding to the identified vertebral mid-height, having a thickness of 0.65 mm, was imported to Analyze (v12, AnalyzeDirect, KS), the contour of the vertebral body inclusive of proximal mid-pedicles segmented, and the corresponding cross-sectional area (CSA) and volumetric BMD computed. To eliminate negative BMD values due to air bubbles within the degassed vertebrae, CT values < 0 indicating air were set to a value of 0. Vertebral strength (Vs) was estimated in all specimens at all levels included in the CT scan using a previously developed regression equation (Mokhtarzadeh et al., 2021), Eq. 1:

$$\mathrm{Vs}\left(N\right)=3524.6\times \mathrm{BMD}\left(g/cm^{3}\right)\times \mathrm{CSA}\left(cm^{2}\right)-267.15$$ (1)

### 2.4 Specimen-Specific Musculoskeletal Modeling for Metastatic Spines

Per our established protocol for creating subject-specific musculoskeletal models for the human spine and torso from *in vivo* CT data (Bruno et al., 2015; Bruno et al., 2017b), a specimen-specific model was created for each of the 11 spines included in this study. The model was created in the OpenSim musculoskeletal modeling software (Delp et al., 2007), and it incorporates the entire thoracolumbar spine, consisting of the 17 individual thoracic and lumbar vertebrae connected by intervertebral joints with three rotational degrees of freedom, as well as the sacrum, pelvis, individual ribs and sternum, lumped head and neck body, upper extremities, and major muscle groups modeled using 552 individual Hill-type musculotendon actuators (Bruno et al., 2015). Specifically, based on the donor’s gender, the appropriate “base” male (Bruno et al., 2015) or female (Bruno et al., 2017a) model was scaled according to the donor’s reported height and weight, which adjusted all body mass and inertial properties, body size, and muscle lengths and attachment points accordingly. Using SpineAnalyzer (Optasia Medical, Cheadle, UK), the donor’s volumetric CT data were processed to obtain mid-sagittal CT projection, the resulting view akin to a clinical mid-sagittal view. The resulting mid-sagittal image was radiographically analyzed to locate six morphometry points around each vertebral body from T4 to L4 (Kim et al., 2011) to compute the vertebral size and intervertebral angles. This vertebral morphometric data were used to adjust the size and curvature of the spine for each specimen-specific model. Trunk muscle size and position parameters were estimated based on donor age, sex, height, and weight using our previously published regressions for predicting muscle parameters for musculoskeletal modeling (Anderson et al., 2012). The muscle groups were adjusted to match the estimated muscle parameters described previously (Bruno et al., 2015). Vertebral compressive and shear loading at each vertebral given body position and external load were estimated by the model output (e.g., weight in hands), thus allowing simulation of various activities.

### 2.5 Simulations of Spinal Loading in Response to Daily Tasks

For this study, four conditions that commonly occur during normal daily activities were simulated, specifically Natural standing (NS) and three conditions involving lifting or holding an object: 1) standing with elbows flexed 90° holding 5-kg weights in each hand (S+W), 2) forward flexion (60°) holding 5-kg weights in each hand (FL+W), and 3) lateral bend (20°) holding a 5-kg weight in the right hand (LB+W). Based on our previous modeling (Mokhtarzadeh et al., 2021), the three lifting activities produced higher LSRs in the lumbar, thoracolumbar, and thoracic regions of the spine, respectively, compared with a wide selection of everyday activities (Mokhtarzadeh et al., 2021).

For the static posture of each activity, overall trunk angles were distributed through the intervertebral joints and pelvis based on reported literature ratios for flexion/extension, lateral bending, and axial rotation, as described previously (Bruno et al., 2015). Static optimization analyses were performed using OpenSim (version 3.3) to estimate muscle activations and forces using a cost function that minimizes the sum of muscle activations cubed. Joint reaction forces were determined and used to estimate the vertebral compressive loading at each vertebral level. Finally, each vertebra’s task-specific LSR was computed by dividing the computed vertebral compressive loading by its CT-based strength estimate.

### 2.6 Musculoskeletal Modeling: Normative Dataset

To compare metastatic spine LSRs with normative LSRs, a sample of 250 individuals from the community-based Framingham Heart Study Multidetector CT Study was used (Hoffmann et al., 2008). Briefly, this dataset comprises an age- and sex-stratified sample from this cohort, including 25 men and 25 women within each of five age groups: 40–49, 50–59, 60–69, 70–79, and >80 years of age (Johannesdottir et al., 2018). Table 2 presents demographic details for the normative dataset. As reported previously (Mokhtarzadeh et al., 2021), CT scans were used to create subject-specific musculoskeletal models of all 250 individuals, similar to the specimen-specific models described above, and the models were used to simulate a variety of static tasks. CT measurements were used to estimate vertebral strength, and LSRs were evaluated as described above. The LSRs from this sample for the four current activities of interest were then used as normative data.

**TABLE 2:** Demographic characteristics of normative subjects included in the study.

Characteristic	Men (*n* = 125)		Women (*n* = 125)	
	Mean (SD)	Range	Mean(SD)	Range
Age(yrs)	64.7 (14.0)	41–88	64.3 (13.6)	43–90
Height (cm)	174.0 (7.3)	159–192	159.9 (6.4)	147–176
Body mass (kg)	85.2 (14.3)	47.2–122.9	70.7 (14.9)	43.5–127.0

The normative dataset was also used to estimate normative LSRs individualized to each specimen for each activity and spinal level, adjusting for age, sex, and body habitus. Specifically, multivariable linear regression was used to predict the LSRs for each activity and spinal level, including age, sex, and bone mass index (BMI) as independent variables (Mokhtarzadeh et al., 2021). Median LSRs were determined in the normative dataset by vertebral level, and the 5th and 95th percentiles were established for each activity examined. Thus, a set of normative LSR estimates matching our specimen characteristics was generated. These outcome medians were used as normative values for comparison in statistical analyses.

### 2.7 Statistical Analysis

Spinal curvature (lordosis, kyphosis) was compared between metastatic spines and the Framingham normative data using Wilcoxon rank-sum tests. The Shapiro–Wilk test was used to evaluate the normality of the distribution of estimated vertebral BMD and strength. The distributions across clinical classifications (NOL, osteosclerotic, osteolytic, mixed) were compared using Kruskal–Wallis tests, followed by Dunn pairwise tests if the global test was significant. Regression methods were used to evaluate the association between the measured strength of the vertebrae and the predicted strength based on two methods (CT-based and homogenized finite element (hFE) modeling predicted). A separate model was fitted for each method. Since the measured strength for seven of the vertebrae exceeded the capacity of the testing system, tobit models were fitted to account for the truncated distribution. R^2^ (coefficient of determination) and AICc (corrected Akaike information criterion) were used to evaluate which prediction method better fit the measured strength (higher R^2^ and lower AICc indicate better fit). For each activity, a Wilcoxon signed-rank analysis that matched each vertebra from a metastatic spine to the activity-specific, level-specific median from the Framingham normative cohort was conducted. This analysis excluded T1, T2, T3, and L5, as these levels were not available in the normative data. In addition, the same analysis was conducted separately for each clinical classification of metastasis type.

A linear mixed-effects regression model was used to identify whether task and clinical classification were associated with LSRs for the metastatic vertebrae. To account for correlated observations among vertebrae from the same spine and factors such as age and sex that may affect LSR, all LSRs from the metastatic vertebrae to their regression-predicted normative values were normalized, thereby adjusting for age, sex, and BMI. The effect of task and clinical classification on this normalized outcome was then evaluated using mixed-effects regression models, with specimen ID as a random effect. The task and clinical classification interaction was not included because our initial modeling found that it was not significant.

## 3 Results

### 3.1 Characteristics of Spine Donors and Normative Dataset Subjects

**FIGURE 2:**
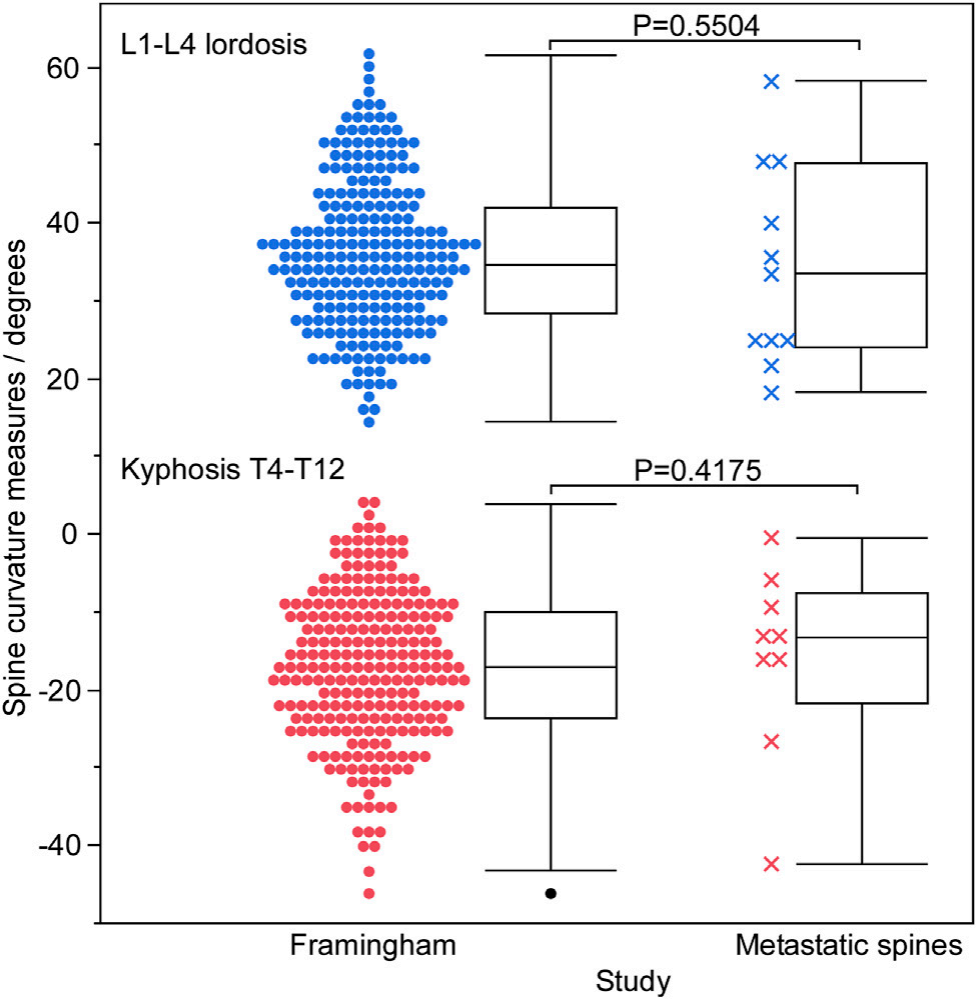
A graphical summary of spinal curvature measured for the Framingham cohort and cadaveric spines using SpineAnalyzer (Optasia Medical, Cheadle, United Kingdom), grouped by lordotic and kyphotic regions. No statistically significant difference was observed between the two groups in either the lumbar or thoracic regions.

The age and height of the 11 spine donors (3 females, 8 males) (Table 1) fell well within the ranges represented by the 125 men and 125 women in the normative dataset (Table 2). The recorded body mass of the donors had a similar range to that of the normative subjects with two exceptions; one female donor’s body mass (40.8 kg) was slightly less than the minimum recorded in the normative data (43.5 kg), while one male donor’s body mass (127.0 kg) was larger than the maximum male body mass in the normative data (122.9 kg). Still, this donor’s body mass was the same as the maximum female body mass in the normative data (127.0 kg). Wilcoxon analysis showed no statistical differences in spinal curvature between the cadaveric spines and the Framingham cohort for either L1–L4 lordosis (*p* = 0.5504) or T4–T12 kyphosis (*p* = 0.4175) (Figure 2).

### 3.2 Metastatic Bone Lesion Classification

Table 3 presents the proportion of the bone lesion type classification identified for each cancer. Univariate analysis revealed CT-derived BMD to be non-normally distributed (Shapiro–Wilk test, *p* = 0.0022–0.008), with osteolytic and osteosclerotic vertebrae exhibiting a bimodal distribution of BMD compared with the unimodal distribution of BMD demonstrated by NOL and mixed lesion vertebrae. Based on the clinical bone metastases classification, median (q1–q3) values of 248.7 (170.7–453.3) g/cm^3^ were exhibited by osteosclerotic vertebrae, 337.9 (240.8–440.3) g/cm^3^ by mixed lesion vertebrae, 180.5 (95.0–324.4) g/cm^3^ by osteolytic vertebrae, and 183.3 (141.1–234.4) g/cm^3^ by NOL vertebrae.

**TABLE 3:** Metastatic spine vertebral classification of lesion type.

Primary	NOL^a^ (%)	Osteolytic (%)	Osteosclerotic (%)	Mixed (%)
Breast (*n* = 3)	20	43	22	16
kidney (*n* = 2)	53	38	6	3
Lung (*n* = 3)	76	8	10	6
Esophageal (*n* = 1)	100			
Prostate (*n* = 2)	3		82	15

a Without radiological evidence of bone metastasis. NOL: no observed lesion.

**FIGURE 3:**
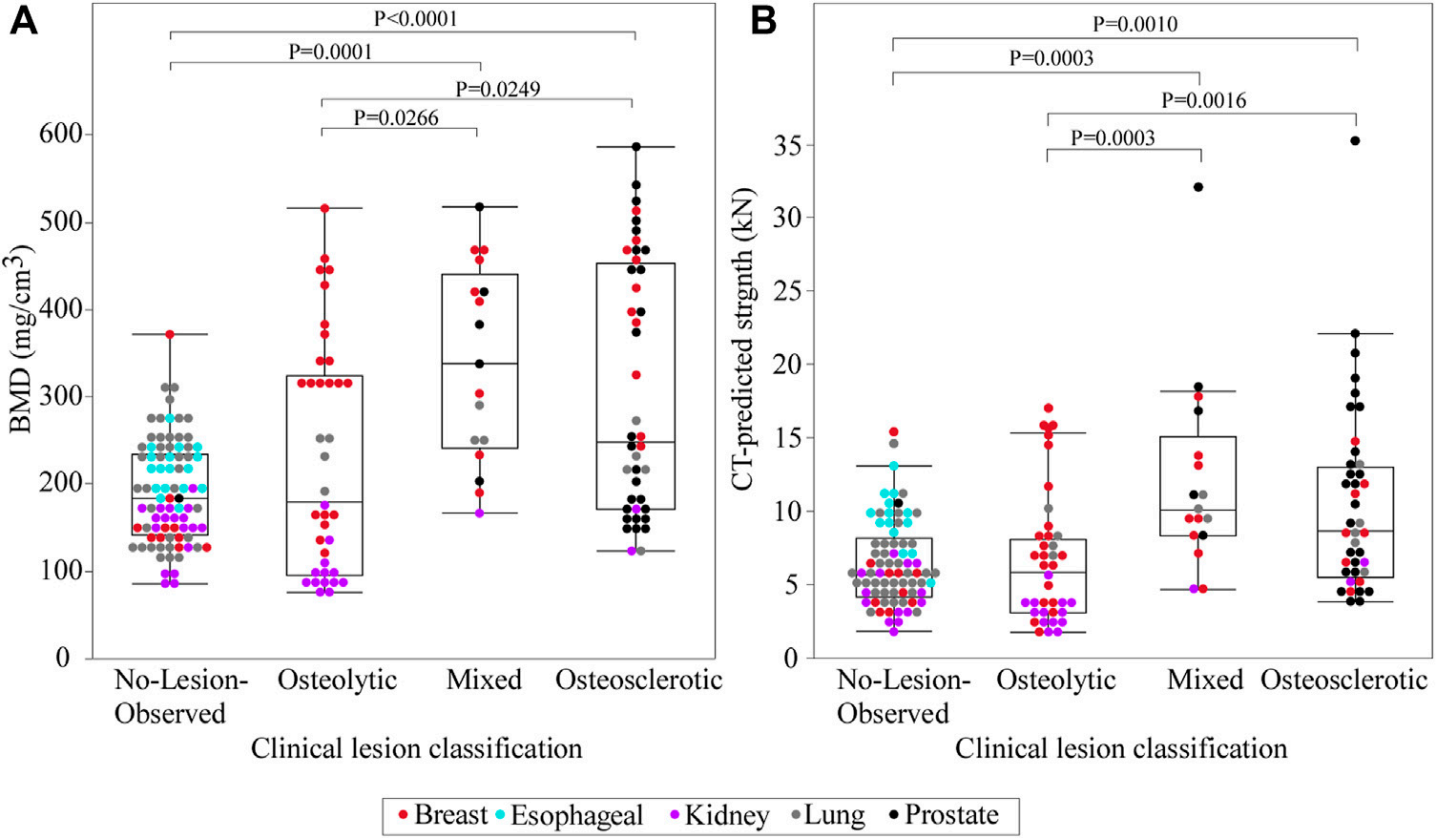
A statistical summary of the CT-estimated bone mineral density (BMD) **(A)** and computed strength **(B)** of the metastatic vertebral levels grouped by bone lesion quality. The boxplot central line indicates the median; the box top and bottom boundaries indicate 25th and 75th percentiles, with whisker lines representing a 95% confidence interval. Vertebrae classified as osteosclerotic and mixed lesions showed significantly higher CT-estimated BMD and computed strength than vertebrae classified as NOL or osteolytic. There was no statistically significant difference in CT-estimated BMD or computed strength between vertebrae classified as NOL and osteolytic or osteosclerotic and mixed lesions.

Non-parametric analysis of variance (Kruskal–Wallis) found that the clinical bone metastases classification significantly affected the estimated BMD (*p* < 0.0001) (Figure 3A). Post-hoc analysis (Dunnes all pairs for joint ranks) revealed that the osteosclerotic and mixed lesion vertebrae had significantly higher BMD values than the osteolytic (*p* = 0.0249 and *p* = 0.0266, respectively) and NOL (*p* = 0.0001 and *p* < 0.0001, respectively) vertebrae. There was no significant difference between the BMD values of the osteolytic and NOL vertebrae (Figure 3A).

### 3.3 Validation of CT Estimates of Vertebral Strength

To validate the protocol for CT estimation of strength, the CT-based prediction of vertebral strength of the 45 vertebrae in the current study was compared with 1) experimentally measured compressive strength (Stadelmann et al., 2020) and 2) vertebrae derived using FEA (Stadelmann et al., 2020).

**FIGURE 4:**
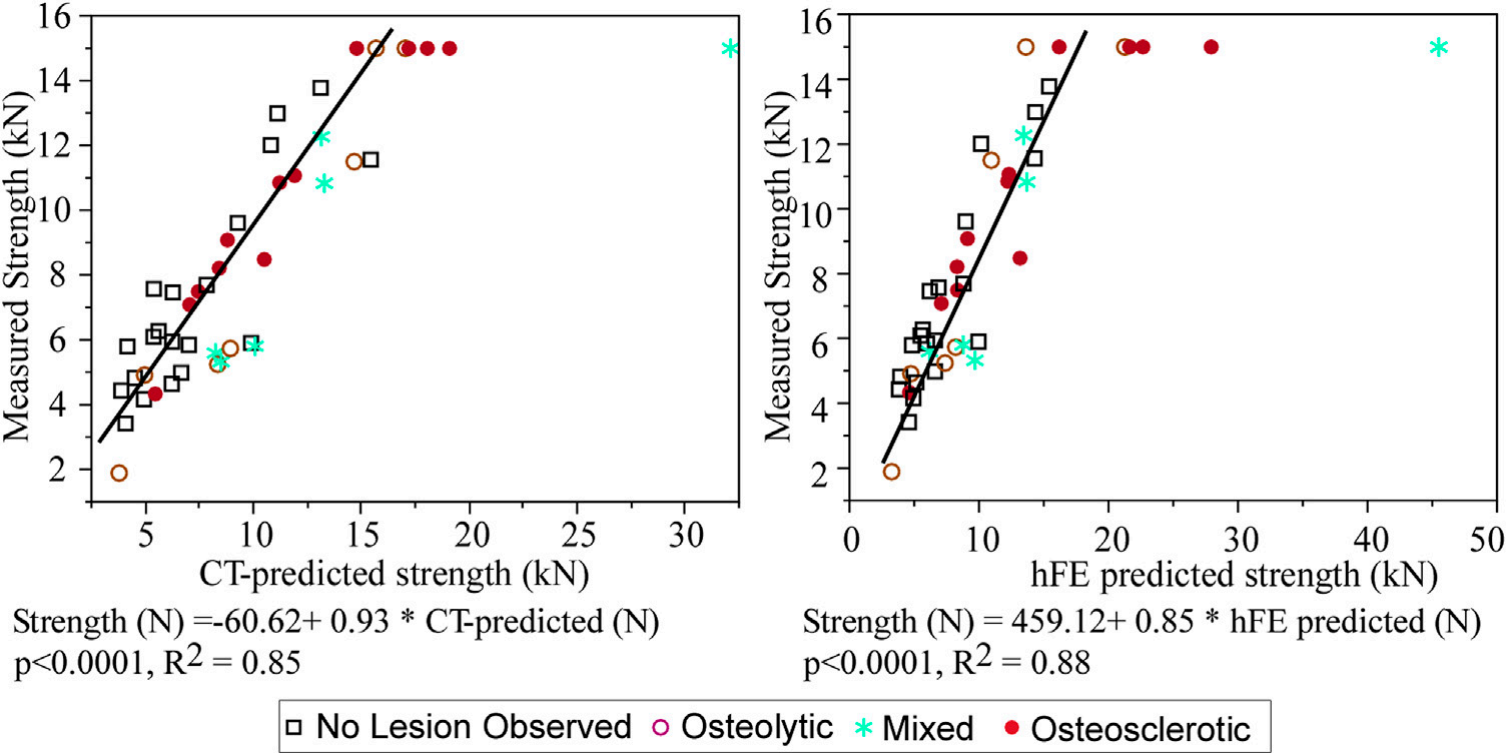
Tobit regression analysis demonstrates that the CT-estimated model predicted strength is strongly associated with the measured vertebral strength, with the model performance agreeing with that obtained independently from FE analysis (Stadelmann et al., 2020).

Seven of the tested vertebrae exceeded the maximum safe force of the MTS built-in load cell (15 kN: model: 662.20D-04, Instron, Canton, MA) (Stadelmann et al., 2020) and were assigned strength = 15 kN. Application of tobit regression (allowing to account for the truncated strength data, i.e., vertebra whose strength is >15 kN) showed that CT-estimated strength explained 85% of the variance in the measured vertebral strength (*p* < 0.0001, R2 = 0.85, AICc = 683.9) (Figure 4). Tobit regression showed FE-computed vertebral strength to explain 88% of the variance in the measured vertebral strength (*p* < 0.0001, R^2^ = 0.88, AICc = 674.2) (Figure 4). Based on AICc, the hFE model better predicted vertebral strength.

### 3.4 CT-Based Estimates of Vertebral Strength

**FIGURE 5:**
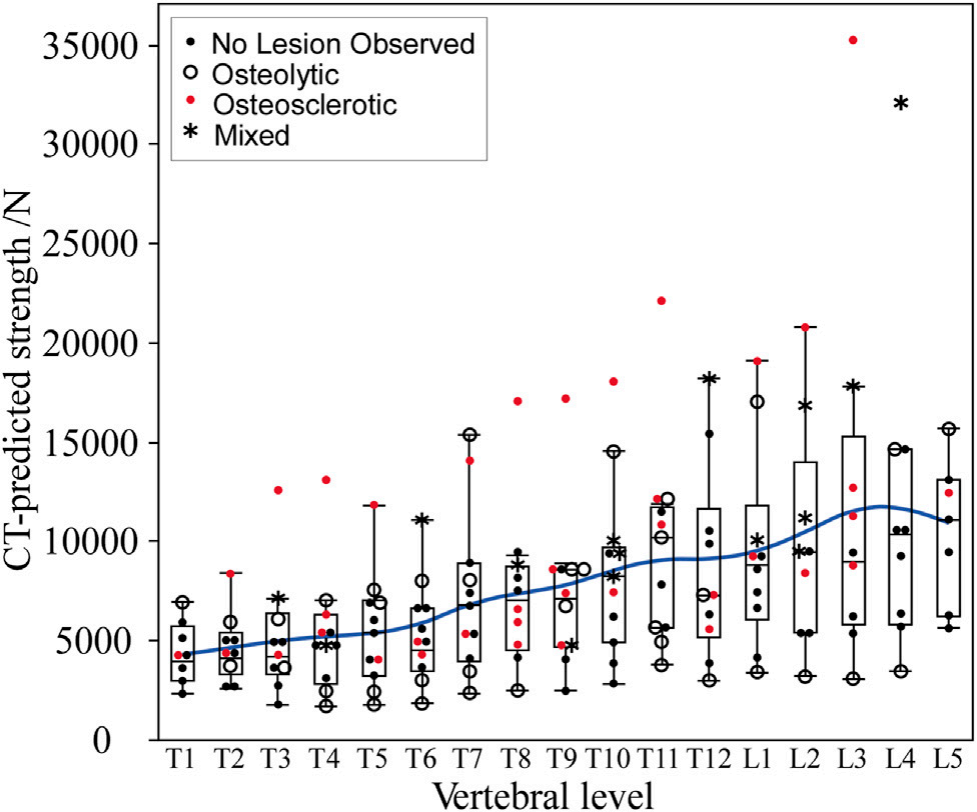
In the metastatic spines, CT-estimated vertebral strength showed a monotonic increase from the upper thoracic to the lower lumbar vertebral levels. The boxplot central line indicates the median; the box top and bottom boundaries indicate 25th and 75th percentiles, with whisker lines representing a 95% confidence interval. Per level, osteolytic vertebrae predominantly formed the lower bounds, with osteosclerotic vertebrae predominantly forming the upper bounds of the estimated strength.

Vertebral strength showed a near monotonic increase from the thoracic to the lumbar region, a median (Q1–Q3) of 4000 (3000–5746) N at T1 to 11,118.9 (6270–13,117) N at L5 (Figure 5). Univariate analysis revealed that within each clinical bone metastases classification (i.e., osteolytic osteosclerotic, mixed, and NOL), the CT-estimated vertebral strength was non-normally distributed (Shapiro–Wilk, *p* < 0.0001). Non-parametric analysis of variance (Kruskal–Wallis) revealed that vertebral grouping based on the clinical bone metastases classification significantly affected the CT-estimated vertebral strength (*p* < 0.0001) (Figure 3B). Post-hoc analysis (Dunnes all pairs for joint ranks) found that the CT-estimated vertebral strength of osteosclerotic vertebrae was significantly higher than that of osteolytic (*p* = 0.0016) or NOL (*p* = 0.0010) vertebrae (Figure 3B). Vertebrae with mixed metastases had significantly higher estimated strength than either osteolytic (*p* = 0.0003) or NOL (*p* = 0.0003) vertebrae (Figure 2B). Surprisingly, we found no significant differences in estimated strength between the NOL and osteolytic vertebrae (Figure 3B).

**FIGURE 6:**
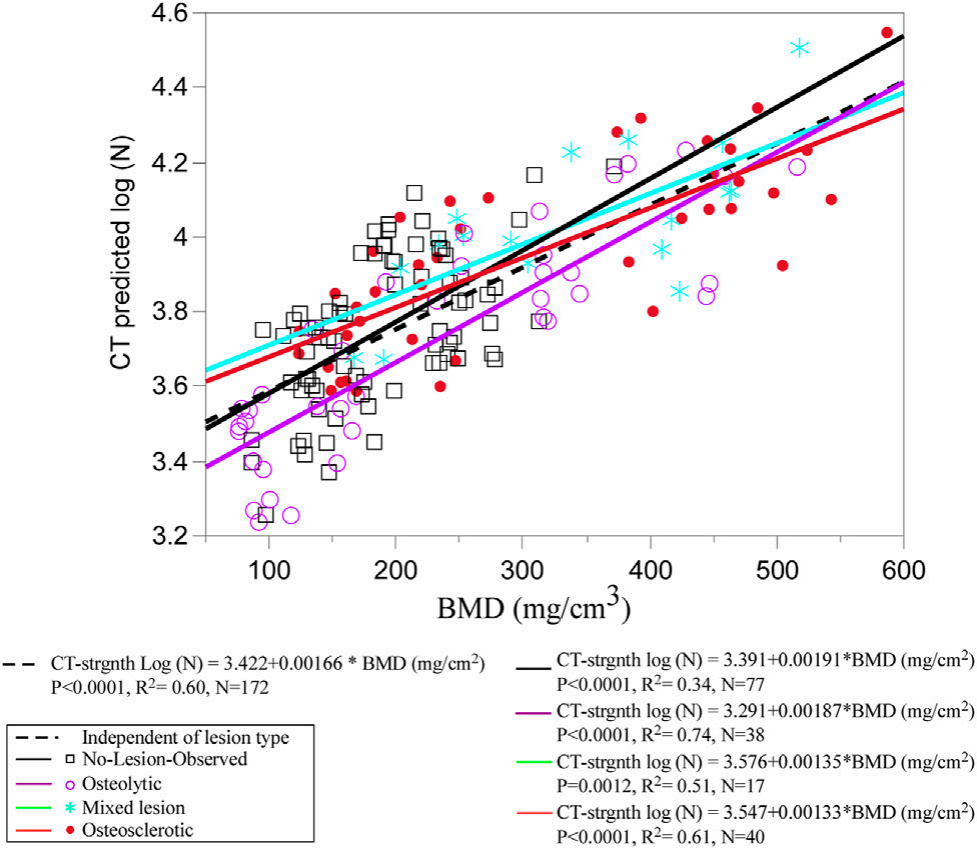
Regression analysis of the pooled data shows CT-estimated BMD to be a strong independent predictor of CT-estimated strength. Grouping by individual metastatic bone type shows that the association was stronger for osteolytic and osteosclerotic bone lesions but weaker for mixed lesions, highlighting the uncertainty in predicting the strength for this type of bone lesion. Surprisingly, the weakest association was observed for the NOL vertebrae, suggesting that vertebral bone in vertebrae without radiographic evidence of bone lesions should not be perceived as free of disease in cancer patients.

Figure 6 presents the association between CT-estimated BMD and log-transformed CT-estimated strength for the pooled data stratified by clinical lesion type. Regression analysis found that CT-estimated BMD values explain 60% of the variance in the CT-estimated strength (*p* < 0.001) (Figure 6). Stratified based on clinical lesion type, CT-estimated BMD values explained 74% of the CT-estimated strength variation in osteolytic vertebrae (*p* < 0.0001), 61% in osteosclerotic vertebrae (*p* < 0.0001), 51% in mixed lesion vertebrae (*p* = 0.0034), and 34% in NOL vertebrae (*p* < 0.0001). A multivariable LMM model with age, sex, race, and BMI as a fixed effect and spine ID as a random effect found that none of the parameters were a significant independent correlate of the CT-estimated strength.

### 3.5 Load-To-Strength Ratios by Activity and Level in Metastatic Spines and Normative Population

**FIGURE 7:**
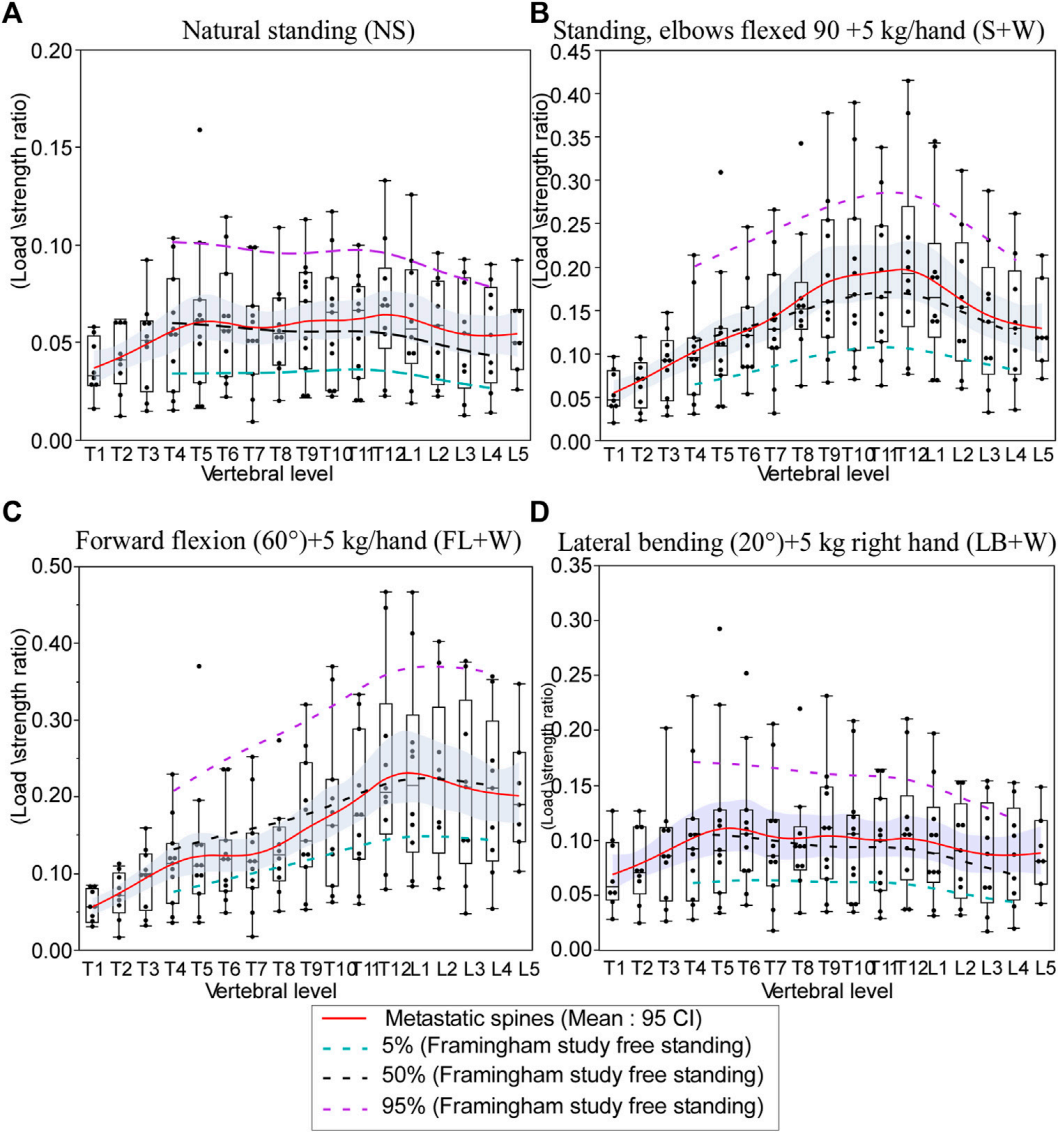
Effect of simulated daily tasks on the change in vertebral compressive. **(A)** Nautral standing, **(B)** standing while holding a weight (elbows flexed 90° with 5 kg in each hand), **(C)** 40° trunk flexion while holding 5 kg in each hand, and **(D)** 20° trunk lateral bending to the right with 5 kg in the right hand. Each subject-specific model was adjusted for height, weight, and spine curvature. The boxplot central line indicates the median; the box top and bottom boundaries indicate 25th and 75th percentiles, with whisker lines representing a 95% confidence interval. The dotted lines represent the 5th percentile (green), median, 50%, (black), and 95th percentile (purple) of the computed LSR values for each activity modeled using the data obtained for the Framingham cohort (Mokhtarzadeh et al., 2021).

Figure 7 presents the metastatic spine LSR median values (T1–L5) computed for each task. For comparison, we present the 5%, 50%, and 95% normative curves computed from the Framingham study for each modeled task. For activities about the sagittal plane (NS, S+W, and FL+W), peak compressive vertebral LSR occurred at the thoracolumbar region (T11–L1), with a secondary local peak at T5-T6 seen for NS and FL+W tasks but not for NS+W task. In response to FL+W, the metastatic spine’s median LSRs showed a significantly altered pattern, with values generally lower (*p* = 0.0121, signed-rank test). However, the decreases were not uniform across the levels of the spine. Compared with 50% LSRs computed for the Framingham cohort, a substantial decrease in values was seen at T4–T10 (a median ranging from −12% to −37%). In contrast, a small increase in values was seen at T12-L1 (a median ranging from 1% to 3%) (Figure 7).

### 3.6 Load-To-Strength Ratios by Activity and Lesion Type Compared With Predicted Normative Values

**FIGURE 8:**
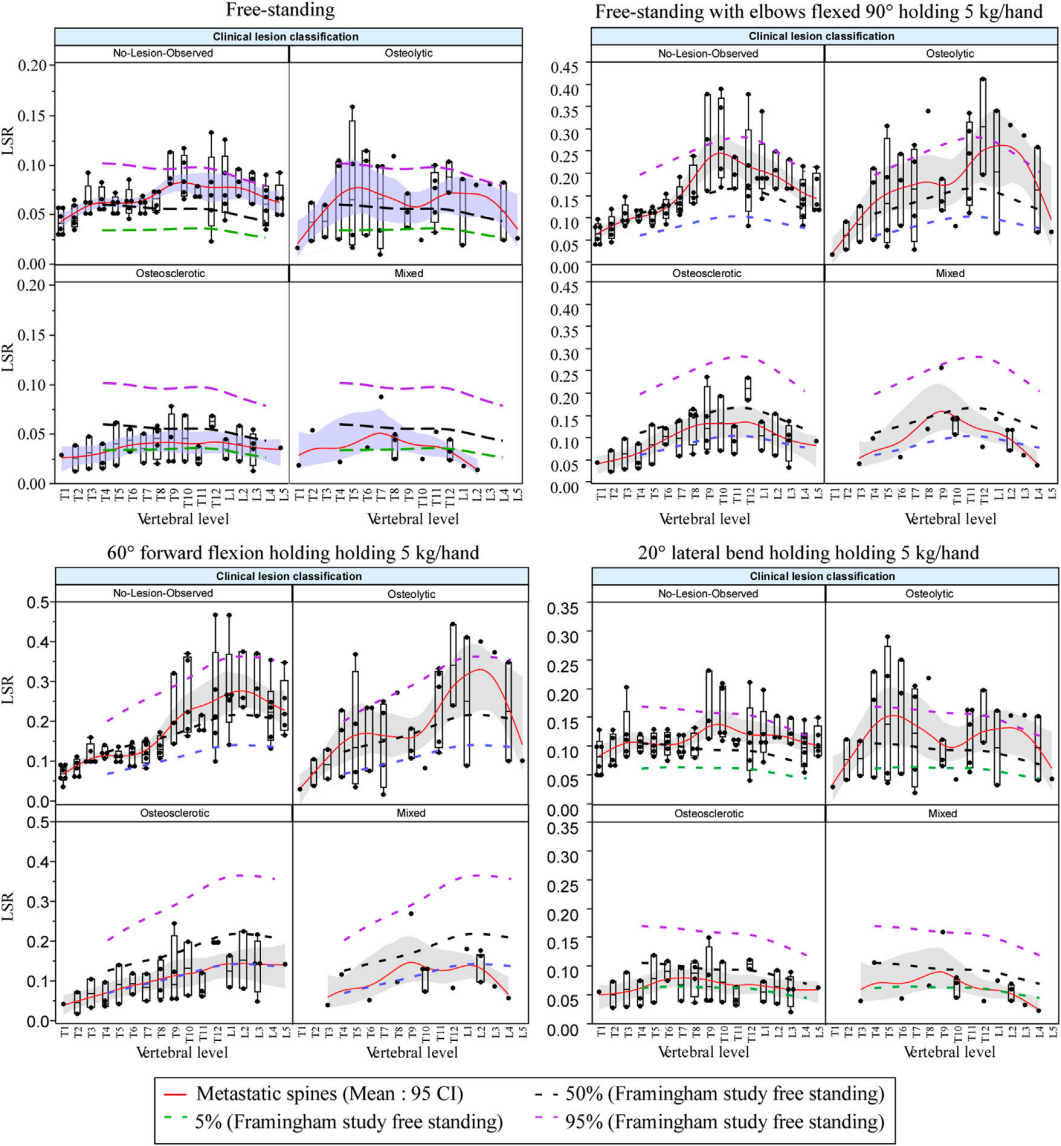
Bone lesion type significantly affected the spine’s LSR for each activity, yielding higher LSRs for osteolytic and NOL vertebrae under simulated natural standing and lateral bending with weight and for osteolytic vertebrae under natural standing with weight compared with the Framingham cohort median (50%) LSRs. Vertebrae with osteosclerotic bone lesions showed lower LSRs for each of the modeled activities than the Framingham cohort median (50%) LSRs, with vertebrae classified as mixed lesions showing similar trends. The boxplot central line indicates the median; the box top and bottom boundaries indicate 25th and 75th percentiles, with whisker lines representing a 95% confidence interval. Dotted lines represent median (50%) LSR 5, and 5th and 95th percentiles derived for each activity examined from the data obtained for the Framingham cohort (Mokhtarzadeh et al., 2021).

Figure 8 presents the effect of the four-bone lesion classifications on the spine LSR curves for each modeled task. The 5%, 50%, and 95% normative curves computed from the Framingham study for each modeled task are presented. The bone lesion classification significantly modified the spine’s LSR for each activity (Figure 8). Compared with the Framingham cohort, vertebrae with osteolytic bone lesions showed higher median LSRs under NS (*p* = 0.0375), S+W *p* = 0.0118), and LB+W (*p* = 0.0111). Similarly, compared with the median (50%) LSRs of the Framingham cohort, NOL vertebrae showed significantly higher LSRs under NS (*p* < 0.0001) and LB+W (*p* = 0.0009). Vertebrae with osteosclerotic bone lesions had lower LSRs under NS (*p* < 0.0001), S+W (*p* = 0.0005), FL+W (*p* < 0.0001), and LB+W (*p* = 0.0002) than the median values for the Framingham cohort. Vertebrae with mixed bone lesions exhibited lower LSRs than the Framingham cohort median values [NS (*p* = 0.0107), S+W (*p* = 0.0107), FL+W (*p* = 0.0301), and LB+W (*p* = 0.0166)], a trend similar to that observed for the osteosclerotic vertebrae. The linear mixed model analysis revealed activity (*p* < 0.0001) and lesion classification (*p* = 0.0032) to be independently associated with normalized LSR (metastatic spine LSR/expected normative LSR). As shown in Table 4, the mean difference for mixed and osteosclerotic vertebrae was below the normative means, while osteolytic levels were above the normative means, and NOL vertebrae were above the normative means except in thoracic levels for FL+W.

**TABLE 4:** Effect of bone lesion type on the difference in activity-based LSRs for thoracic and lumbar metastatic spines, compared with normative data derived from the Framingham Heart Study.

Activity	Metastatic bone lesion classification			
	No lesion observed (%)	Osteolytic (%)	Mixed (%)	Osteosclerotic (%)
Thoracic				
Free standing	7.08	4.60	−40.03	−22.65
Standing + 5 kg weight	6.07	7.16	−36.50	−32.02
Forward Flexion + 5 kg weight	−12.70	1.13	−44.59	−41.20
Lateral bending + 5 kg weight	14.47	18.14	−46.18	−22.46
Lumbar				
Free standing	47.64	78.35	−48.06	−16.24
Standing + 5 kg weight	30.93	65.62	−45.90	−37.38
Forward Flexion + 5 kg weight	19.95	60.80	−48.43	−40.68
Lateral bending + 5 kg weight	43.59	106.94	−41.79	−21.35

All values are presented as a percent difference between means.

## 4 Discussion

Predicting the risk of PVF, a common complication associated with vertebral bone metastases (Prasad and Schiff, 2005), forms a critical determinant in managing this patient cohort (Siegel et al., 2012; Yao et al., 2017). PVF occurs when the disease burden diminishes the strength and anatomical integrity of the affected vertebrae, rendering the lesioned vertebrae unable to withstand the loads applied. Given the absence of a quantitative assessment for PVF risk in the clinical setting (Yao et al., 2017), better fracture risk prediction represents a significant unmet medical need (Galasko, 1986; Aebi, 2003; Bartanusz and Porchet, 2003; Groot et al., 2006). This study provides the first application of the LSR concept to vertebrae with metastatic disease and suggests that these pathologic vertebrae differ from normal spines in important ways. The finding of lower than normal LSRs in osteosclerotic vertebrae and higher than normal LSRs in osteolytic vertebrae corresponds to clinical expectations, as these vertebrae are understood to have a lower and higher risk of PVF (Weber et al., 2011), respectively. Surprisingly, higher LSRs were found in NOL vertebrae than in the normative dataset, suggesting that fracture risk could be increased in patients with spine metastases even at levels where lesions have not been identified radiologically. It is also important to note that LSRs were well above the 95th percentile for the normative dataset for several vertebrae identified with osteolytic bone metastases. This difference could indicate vertebral levels at a higher than normal risk of PVF, a possibility that should be evaluated in future studies examining PVF risk in patients.

Assessment of LSR requires estimating vertebral strength and task-associated loading. This study evaluated a CT-based method, initially developed in a Framingham Heart Study cohort sample with no evidence of spinal bone metastases (Christiansen et al., 2011; Samelson et al., 2012), to estimate the compressive strength of 45 human vertebrae obtained from spine specimens with metastatic disease (Stadelmann et al., 2020). Comparison with strength prediction obtained independently based on the FE approach (Stadelmann et al., 2020) demonstrated that the CT-based model predicted vertebral strength in close agreement with the FE strength prediction (Stadelmann et al., 2020). This finding indicates that the CT approach is generalizable for estimating pathologic vertebral compressive strength. Based on this validation, BMD and strength values of 172 cadaveric vertebrae from 11 donors with solid bone metastases from breast, esophageal, kidney, lung, and prostate cancer were estimated. Of note, 37% of the osteolytic vertebrae, obtained from donors with kidney, lung, and breast cancer, had BMD < 140 mg/cm^3^, of which 15 had BMD < 85 mg/cm^3^. Such values suggest that the osteolytic vertebrae are osteopenic (Zysset et al., 2015) and highlight the loss of bone fraction (Hojjat et al., 2011; Hojjat et al., 2012; Burke et al., 2017) and mineral content (Burke et al., 2017; Stadelmann et al., 2020) associated with the infiltration of osteolytic bone metastasis in vertebral bone (Nazarian et al., 2008; Burke et al., 2016; Burke et al., 2017). In agreement with their radiological appearance, 45% of the osteosclerotic vertebrae from prostate and breast cancer donors demonstrated BMD with a mean (SD) of 462.9 (56.4) mg/cm^3^, as shown in previous studies; this is the result of extensive deposition of bone mineral content, a high degree of remodeling, and increased thickness of bone trabeculae in bone regions with osteosclerotic metastasis (Sone et al., 2004; Tamada et al., 2005; Nazarian et al., 2008; Stadelmann et al., 2020). However, as can be observed from Figure 3, 50% of the osteolytic vertebrae exhibited markedly higher BMD values [mean (SD) of 353.5 (78.7) mg/cm^3^] than value estimated for 42% of vertebrae classified as osteosclerotic [190.5 (43.0) mg/cm^3^] or the entire group classified as NOL [190.5 (58.6) mg/ Q19 cm^3^] vertebrae. This overlapping of values highlights the limitation of applying the categorical, highly subjective, radiological classification to delineate the remarkable spectrum of changes in bone density and architecture affected by bone metastases.

In agreement with their radiographic appearance, osteosclerotic and mixed lesion vertebrae exhibited significantly higher CT-derived strength values than osteolytic or NOL vertebrae. However, no significant difference in CT-estimated strength was detected between NOL and osteolytic vertebrae, a finding likely caused by the significantly higher coefficient of variation (COV) of strength of the osteolytic vertebrae than the NOL vertebrae (86.5% vs. 72.2%, F-test two-sided, *p* = 0.0025). At present, little information is available on the effect of metastatic lesions on the variability of strength values in human spines. A recent study of eight patients with osteolytic bone metastases by Costa et al. (2019) estimated vertebral strength from clinical CT using FE computation; they reported that osteolytic vertebrae exhibited strength COV at 43%, a value markedly lower than that found in this study. However, the study by Costa et al. provided no information regarding the patient’s primary cancer, making direct comparisons difficult. In agreement with our previous studies (Stadelmann et al., 2020; Alkalay et al., 2021), univariate regression analysis found the association between the CT-estimated BMD and vertebral strength to be high in osteolytic vertebrae (R^2^ = 0.71), moderate in osteosclerotic vertebrae (R^2^ = 0.53), and weak in mixed lesion vertebrae (R2 = 0.44). The finding of a low association between CT-estimated BMD and strength in vertebrae with mixed lesions, of which strength was shown to be determined by the extent of osteolytic lesions within the bone network (Hojjat et al., 2012; Alkalay et al., 2021), may explain the clinicians’ increased uncertainty when evaluating the degradation of vertebral strength (“instability”) in vertebrae with mixed metastases. Poor strength prediction (R^2^ = 0.33) was observed when evaluating the NOL vertebrae. Given the multivariable model finding of neither age, sex, race, nor BMI as non-significant predictors of the CT-estimated strength and the minor differences in the material modulus of bone obtained from osteolytic and osteosclerotic vertebrae (Burke et al., 2018; Stadelmann et al., 2020), it is likely that the low BMD values of the NOL vertebrae, indicating a low bone fraction, significantly contributed to the observed poor association (Nazarian et al., 2008; Stadelmann et al., 2018; Alkalay et al., 2021). Importantly, this observation indicates that when clinically evaluating vertebral levels with no apparent radiological evidence of bone metastases, such vertebrae should not be assumed to be free of disease and thus expected to structurally function and have a mechanical strength similar to non-pathologic vertebrae. In agreement with Costa et al. (2019) and based on our previous work (Burke et al., 2018; Stadelmann et al., 2020), this study data suggest that the strength of pathologic vertebrae is driven predominantly by spatial variation in the density fraction of the pathologic bone and the geometrical properties of the affected vertebra. Incorporating measures of vertebral bone density and vertebral CSA may provide a quantitative estimate of the mechanical strength of pathologic vertebrae with a wide range of metastatic lesions.

The LSR differences from normative values determined for osteolytic and NOL vertebrae are similar to differences previously reported concerning OVFs. Several studies of OVFs have reported higher LSRs in older adults with prevalent (Duan et al., 2006; Melton et al., 2007; Melton et al., 2010; Anderson et al., 2014; Mokhtarzadeh et al., 2021) or incident (Wang et al., 2012; Kopperdahl et al., 2014) fractures, compared with controls. The most commonly reported LSR has been at a lumbar spine level (L1 or L3) for 90° forward flexion while holding 10 kg (Melton et al., 2007; Melton et al., 2010; Wang et al., 2012; Kopperdahl et al., 2014). In one of these studies (Wang et al., 2012), which reported the LSR as a significant predictor of incident osteoporotic vertebral fracture, the LSR was 0.58 ± 0.26 in cases and 0.36 ± 0.12 in controls. Moreover, the mean LSRs in fracture cases were 21–61% higher than the mean LSRs in control groups in these studies. Only our recent study of the Framingham Heart Study cohort (Mokhtarzadeh et al., 2021), used here as the normative dataset, has examined LSRs throughout the spine and for a wide variety of activities. In that study, it was found that for FL+W, the LSRs were 13%–26% higher for subjects with prevalent vertebral fractures than for those without, specifically at levels T8–L4. Similarly, the LSRs were 17%–35% higher for subjects with prevalent fractures, specifically at levels T5, T6, and T8–L4 for standing with weight. However, no significant effects of prevalent fracture were found for lateral bending with weight. Interestingly, similar effects of lesion type were found in the current study for all three of these loading scenarios. The LSRs for NOL and osteolytic vertebrae were 20–107% higher than normative values in the lumbar spine, similar to or greater than the differences previously reported for prevalent and incident OVFs. These effects were more equivocal in the thoracic spine, with LSRs ranging from −13% to 18%.

On the other hand, the LSRs for mixed (−48% to −37%) and osteosclerotic (−41% to −16%) levels were more uniform among the various activities and in both thoracic and lumbar regions. Altogether, prior studies suggest that LSR may be predictive of vertebral fracture and that examining various loading scenarios is important, as they may produce differing outcomes. However, it is important to note that previous studies have only examined LSR in relation to a vertebral fracture occurring at any vertebral level. In the context of PVF, it is crucial to assess risk on a level-specific basis. The current results suggest that LSRs from vertebrae classified as osteolytic or NOL are on average higher than healthy normative values, while those classified as mixed or osteosclerotic on average have lower LSRs. The size of these differences is likely clinically meaningful in terms of vertebral fracture risk. Our findings regarding vertebrae with mixed lesions should be carefully interpreted. In the SINS protocol, vertebrae containing mixed bone lesions are assigned a lower risk of fracture compared to vertebrae containing osteolytic lesions, while considered at a higher risk than vertebrae containing osteosclerotic bone lesions. However, with the higher ambiguity in classifying these bone lesions, the assigned risk reflects clinicians' increased uncertainty in evaluating the effect of mixed bone metastases on the degradation of vertebral strength (“instability”) in vertebrae with mixed metastases. Although osteoblastic vertebrae are likely to be more robust than mixed or osteolytic vertebrae, our previous work (Alkalay et al., 2021) highlighted the importance of osteolytic regions within the osteoblastic bone network for determining the failure of mixed lesion vertebrae. We are currently investigating incorporating spatial measures of the structural heterogeneity associated with mixed bone lesions to better predict vertebral strength in lesion classification. Future studies of patients with spine metastases are needed to examine whether LSR analysis can predict level-specific fracture risk.

This study has several limitations that should be noted. The analysis was limited to specimens from only 11 spines. Most spines contained multiple lesion types, allowing a reasonable analysis of the effect of lesion classification, but the small numbers preclude any meaningful comparisons based on primary cancer or other patient characteristics. CT-based assessment of apparent BMD is sensitive to the spatial image resolution (partial volume effects) and degree of CT noise determined by the physical parameters of the imaging (CT tube voltage and current, reconstruction kernel, and patient girth) (Solomon et al., 2013; Malkus and Szczykutowicz, 2017), which may affect the relationship between the calibration phantom and CT HU values. The degree to which these factors affect the derivation of vertebral strength for cancer patients *in vivo* is unknown. The CT model’s prediction of vertebral strength is isolated to the prediction of the vertebral body. This prediction does not consider the contribution of the posterior elements to the transfer of loading between vertebral levels, the degree of which is dependent on the interaction between the geometry and spinal curvature of the spinal region-based facet joints (Adams and Hutton, 1980) and the degenerative condition of the intervertebral disc (Shirazi-Adl et al., 1984; Fujiwara et al., 2000; Zirbel et al., 2013). Furthermore, the effect of the intervertebral disc degenerative state on the strength of the vertebral body was not considered (Maquer et al., 2014; Maquer et al., 2015). Hence our analysis is limited to the effect of LSR on spinal failure initiated in the vertebral body.

The use of musculoskeletal modeling has several limitations that should be noted. The creation of specimen-specific musculoskeletal models utilized the spinal curvature observed in the cadaveric spines, which may or may not be the same as the curvature if observed *in vivo*.

Nonetheless, the curvatures of the specimens were well within the curvatures observed *in vivo* in the normative dataset, suggesting that this was not a likely source of differences between the groups.

The specimen-specific models also lacked measurements of trunk muscle, which are included when creating subject-specific models from *in vivo* CT data, as in the Framingham cohort. We have previously reported that the RMS error of loading from scaled models that lacked both curvature and muscle data ranged from 8% to 15% (Bruno et al., 2017b). We would not expect random errors in loading estimates up to 15% RMSE to notably change our results and conclusions, as the magnitude of differences reported was often much greater than 15%.

Only compressive loads were considered, as only valid estimates and measurements of compressive strength were available, but shear and bending loads may play important roles in PVF. Thus, consideration of these outcomes is an important area for future study. The application of standardized static loading scenarios may not represent a particular individual’s posture or motion should such a task be performed. Moreover, static poses likely underestimate the loading under comparable dynamic scenarios, and the current study does not examine the potential effects of geometric or kinematic asymmetries, which may be important in patients with spinal metastases. However, despite these limitations, standardized loading allowed a direct comparison to normative values calculated for the same scenarios, a significant strength of this study. Overall, the application of musculoskeletal modeling in this dataset of metastatic specimens is highly novel and provides new insights into the biomechanics of metastatic vertebrae and PVF.

In conclusion, this analysis demonstrates that metastatic spines may differ from population norms in LSR and that clinical lesion classifications modify LSR. The results thus support possible applications in evaluating the risk of PVF during various loading scenarios and potentially reducing risk by targeted activity modifications. Future clinical studies should evaluate whether LSRs predict PVF in patients with metastatic disease of the spine.

## Data Availability

The raw data supporting the conclusion of this article will be made available by the authors, without undue reservation.
